# Supplementary material for: Massively Parallel CRISPR-Cas9 Knockout Screening in Sheep Granulosa Cells for FSH Response Genes
Source: Animals (Basel). 2024 Mar 14;14(6):898. doi: 10.3390/ani14060898 (PMC10967520; doi:10.3390/ani14060898)

Table S2 CRISPR Cas9 knockout cell library sgRNA gene fragment next generation sequencing data

Sample	Raw Reads	Clean Reads	Raw Base(G)	Clean Base(G)	Effective Rate(%)	Error Rate(%)	Q30(%)	GC Content(%)
chr2	8129840	8076060	1.22	1.21	99.34	0.03	92.88	32.83
chr3	8389142	8340282	1.26	1.25	99.42	0.03	92.06	32.67
chrX	11951826	11929060	1.79	1.79	99.81	0.03	92.53	32.57
chr2-FSH	6907844	6847698	1.04	1.03	99.13	0.03	92.79	32.85
chr3-FSH	8766024	8578208	1.31	1.29	97.86	0.03	92.89	33.02
chrX-SH	7976278	7946230	1.20	1.19	99.62	0.03	97.77	93.22

Table S4. RNA concentration in granulosa cells RNA-seq

Sample	RNA concentration (ng/uL)	volume (uL)	Total RNA (ug)	Integrity value （260/280）
Ctr_1	376	35	13.16	9.6
Ctr_2	219	35	7.665	9.6
Ctr_3	428	35	14.98	9.6
Low_1	348	35	12.18	9.7
Low_2	391	35	13.685	9.5
Low_3	492	35	17.22	9.6
High_1	371	35	12.985	9.5
High_2	497	35	17.395	9.6
High_3	650	35	22.75	9.6

Table S5. Statistics of tissues transcriptome filtered data

Sample	Raw Reads (bp)	Clean Reads (bp)	Clean reads Q30 (%)	GC Content （%）	Overall alignment rates (%)
Ctr_1	49264210	47332760	93.93	50.36	90.93
Ctr_2	50123360	47863106	93.39	49.1	91.83
Ctr_3	42965336	39923276	93.54	52.48	87.04
Low_1	43878046	40299282	93.63	51.91	88.45
Low_2	43468144	40244778	93.83	52.16	88.45
Low_3	44924754	41589454	93.70	51.83	88.84
High_1	39748094	37661634	93.87	50.93	89.99
High_2	45100314	43365220	93.95	52.26	89.60
High_3	42889810	40495944	93.43	51.25	89.57

Figure S1. sgRNA reads sequence distribution and frequency accumulation

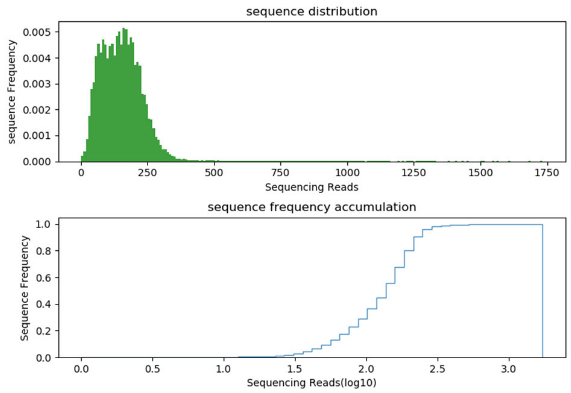

Supplement: Supplementary file 1 [file animals-14-00898-s001.zip › animals-2853295-supplementary/Supplementary Files/Summplement.pdf]
